# Genetic differences among *Moraxella bovis* and *Moraxella bovoculi* isolates from infectious bovine keratoconjunctivitis (IBK) outbreaks in southern Brazil

**DOI:** 10.1590/1678-4685-GMB-2018-0380

**Published:** 2020-05-29

**Authors:** Helena Brocardo Comin, Robert Domingues, Emanuelle Baldo Gaspar, João Rodrigo Gil De Los Santos, Fernando Flores Cardoso

**Affiliations:** 1 Universidade Federal de Pelotas, Programa de Pós-Graduação em Zootecnia, Pelotas, RS, Brazil.; 2 Embrapa Pecuária Sul, Bagé, RS, Brazil.; 3 Universidade Federal de Pelotas, Departamento de Zootecnia, Pelotas, RS, Brazil.

**Keywords:** Beef cattle, DNA sequencing, genetic diversity, RAPD

## Abstract

The objective of this study was to evaluate the genetic diversity of *Moraxella bovis* and *Moraxella bovoculi* bacteria isolated from infectious bovine keratoconjunctivitis (IBK) outbreaks in the state of Rio Grande do Sul, Brazil. The genetic diversity among *Moraxella* spp. was evaluated by RAPD-PCR, JWP1-JWOPA07-PCR, ERIC-PCR and by sequencing the 16S-23S intergenic regions. Based on the dendrogram, two genetically differentiated clades were observed; 14 isolates were classified as *M. bovis* and 17 as *M. bovoculi*. Genetic distances between the *M. bovis* samples ranged from 0.0379 to 0.4285, while for *M. bovoculi* the dissimilarities ranged from zero to 0.7297. Alternatively, based on sequencing analyses of the 16S-23S intergenic region, *M. bovis* and *M. bovoculi* isolates were grouped into the same two different clades, but it was not possible to differentiate between isolates within clades. PCR techniques were demonstrated to be a satisfactory tool to unravel the genetic variability among *Moraxella* spp., while sequencing of the 16S-23S intergenic region was only able to differentiate two species of the *Moraxella* genus. Despite sampling geographically close regions, we demonstrate considerable genetic diversity in *M. bovis* and *M. bovoculi* strains and genetically distinct *M. bovis* strains co-infecting the same animal.

Infectious bovine keratoconjunctivitis (IBK) is the most important ocular disease in bovine production (Snowder *et al.*, 2005). The main observed symptoms are: intense lacrimation, photophobia, conjunctival swelling, opacity in the center of the cornea and ulceration (Postma *et al.*, 2008). Despite its low mortality, this disease is characterized by high morbidity, as it can affect up to 80% of the herd (Postma *et al.*, 2008). It causes significant economic and productive losses due to the reduction in weight gain, decrease in milk production, expenses with repetitive treatments, commercial devaluation, and eventual discard of animals that present severe and permanent ocular sequelae (George, 1990; McConnel *et al.*, 2007). There are no estimates of the economic impact of this disease in the last 20 years.

The etiologic agent of IBK is *Moraxella bovis*, a Gram-negative bacterium, widely disseminated and highly contagious (McConnel *et al.*, 2007). Another *Moraxella* species, *M. bovoculi*, has been frequently isolated from ocular and nasal secretions of animals affected by IBK (Angelos *et al.*, 2007; Sosa and Zunino, 2012; Karthik *et al.*, 2018). However, the role of this species as IBK causal agent is not fully defined, since the experimental infection with this species failed to provoke the disease (Angelos, 2015).

IBK was diagnosed in most Brazilian states and is widely disseminated in the southern region of Rio Grande do Sul state (Turnes, 2001), mainly affecting taurine bovines (Conceição and Turnes, 2003). It is a seasonal disease, with a worldwide distribution and, generally, highly prevalent (McConnel *et al.*, 2007). It affects animals of all ages, but young animals are generally more susceptible (Killinger, 1977).

Therapeutic and prophylactic conduct against IBK includes the use of antimicrobials and vaccines. However, prophylaxis is generally hampered by low vaccine efficacy (O’Connor *et al.*, 2011; Cullen *et al.*, 2017; O’Connor *et al.*, 2019). This situation can be explained by the genetic and antigenic variation between *M. bovis* strains and the presumed presence of other microorganisms involved in this disease (Brown *et al.*, 1998; Kowalski *et al.*, 2017).

The development of molecular techniques allowed the characterization of genetic differences among bacterial isolates. Bacterial genetic variability can be estimated by comparing differently sized DNA fragments, generated from PCR amplification with the use of random primers (Saiki *et al.*, 1988), or by sequencing the internal transcribed spacer (ITS) (Cieslinska *et al.*, 2015; Tokajian *et al.*, 2016).

The main DNA fingerprinting techniques to assess genetic variability among bacteria are PCR-based ones, such as random amplification of polymorphic DNA (RAPD), and repetitive enterobacterial intergenic consensus (ERIC)-PCR. The PCR-based techniques require no specific knowledge of the DNA sequence of the target organism, resulting in several anonymous, not previously determined, but reproducibly amplified fragments (Bowditch *et al.*, 1993). RAPD is based on randomic amplification of DNA with decamer primers (Shekhawat *et al.*, 2019). ERIC-PCR is similar to RAPD, but with larger primers (Hulton *et al.*, 1991). These techniques were successfully used to verify the genetic variability between *M. bovis* and *M. bovoculi* strains in studies focused on the epidemiology of IBK (Prieto *et al.*, 1999; Conceição *et al.*, 2004; Sosa and Zunino, 2013). On the other hand, DNA sequencing of ITS is valuable for species identification (Cieslinska *et al.*, 2015; Tokajian *et al.*, 2016), due to the high variability in this region. Additionally, it can be used to trace inter- and intraspecific genetic variability (Spacov *et al.*, 2006).

The objective of the present study was to characterize the genetic diversity of *M. bovis* and *M. bovoculi* isolates from IBK outbreaks between 2015 and 2017 from five municipalities in Rio Grande do Sul, Brazil, by RAPD-PCR, JWP1-JWOPA07-PCR, ERIC-PCR, and sequencing the ITS.

The study was approved by Ethics Committee on Animal Experimentation of the Federal University of Pelotas (approval number 2328-2017, approved at 02 October 2017). Five non-vaccinated herds, with most of the cows displaying signs of IBK at the time of collection were enrolled in the study. Around 18 animals per herd were sampled, although the number of animals in each herd was 120 on average. Four samples per animal were collected, totalizing 360 samples, from 90 cattle with initial clinical symptoms of IBK. Swabs from both eyes and nostrils were collected, immediately seeded on blood agar, incubated at 37 °C, and 24 h later, small, white and β-hemolytic colonies were re-seed on blood agar for further characterization. From 360 samples, 28 *Moraxella* spp. were isolated and characterized as *M. bovis* and *M. bovoculi* by PCR-RFLP, according to Angelos and Ball (2007). Additionally, three other strains, previously isolated and identified as *M. bovis* and *M. bovoculi* (Taim-1; Taim-2 and Taim-3) were added to the study. All samples were collected in Rio Grande do Sul state, Brazil, totalizing 31 samples (Table 1).

**Table 1:** Molecular characteristics of *Moraxella* spp. isolated from IBK, in five municipalities of Rio Grande do Sul state, Brazil.

N°	Isolates	Origin	Surge	Homology
1	151 OE	Alegrete	2016	*M. bovis*
2	Taim – 2	Rio Grande	2016	*M. bovis*
3	171 OD	Alegrete	2016	*M. bovis*
4	171 OE	Alegrete	2016	*M. bovis*
5	324 OE	Alegrete	2016	*M. bovis*
6	6213 OD	Dom Pedrito	2016	*M. bovis*
7	391 OD	Alegrete	2017	*M. bovis*
8	391 OE	Alegrete	2017	*M. bovis*
9	976 OD	Alegrete	2016	*M. bovis*
10	128 OE	Alegrete	2016	*M. bovis*
11	6587 ND	Dom Pedrito	2017	*M. bovis*
12	6587 OD	Dom Pedrito	2017	*M. bovis*
13	6219 OD	Dom Pedrito	2016	*M. bovis*
14	6456 OE	Dom Pedrito	2016	*M. bovis*
15	666 OD	Alegrete	2016	*M. bovoculi*
16	Taim – 1	Rio Grande	2016	*M. bovoculi*
17	Taim – 3	Santa Vitória do Palmar	2016	*M. bovoculi*
18	6052 OE	Dom Pedrito	2015	*M. bovoculi*
19	1368 OD	Dom Pedrito	2016	*M. bovoculi*
20	1138 OE	Dom Pedrito	2017	*M. bovoculi*
21	1192 OD	Dom Pedrito	2017	*M. bovoculi*
22	281 OD	Uruguaiana	2017	*M. bovoculi*
23	5623 OE	Dom Pedrito	2015	*M. bovoculi*
24	330 NE	Alegrete	2016	*M. bovoculi*
25	259 OD	Dom Pedrito	2016	*M. bovoculi*
26	29 OE	Alegrete	2016	*M. bovoculi*
27	1295 OD	Dom Pedrito	2016	*M. bovoculi*
28	1362 OE	Dom Pedrito	2016	*M. bovoculi*
29	1213 NE	Dom Pedrito	2017	*M. bovoculi*
30	120 ND	Uruguaiana	2015	*M. bovoculi*
31	120 OD	Uruguaiana	2015	*M. bovoculi*

OD = Right eye / OE = Left eye / ND= Right nostril and NE = Left nostril.

The extraction of genomic DNA was based on the protocol developed by Ausubel *et al.* (2003). The concentration and purity of DNA samples were evaluated by nano-spectrophotometry (NanoDrop ND-2000), and DNA integrity by 1% agarose gel electrophoresis.

Initially, the genetic diversity was evaluated by RAPD. For primer selection, a set of 20 decamer primers with arbitrary sequence (OPA-01 to OPA-20 – Operon Technologies, Alameda, USA) was tested with two *M. bovis* and two *M. bovoculi* samples. The primers that produced a higher number of analyzable amplicons, i.e. producing thick and strong bands, were chosen for the reproducibility of the technique. Amplification reactions used the parameters of Domingues *et al.* (2011). DNA fragments from PCR amplification were loaded on a 1.5% agarose gel and submitted to electrophoresis in TBE buffer, stained with ethidium bromide, and photo-documented on UV light using the Imaging Systems (UVITEC). This initial step allowed to choose seven primers that were then used to analyze all the samples.

The degree of genetic diversity among *Moraxella* spp. was also investigated with primers the JWP1-JWOPA07 and ERIC, according to Sosa and Zunino (2013), who had used them to assess the genotypic diversity of the isolates of *M. bovis* and *M. bovoculi*, from Kansas (USA) and Uruguay. The primers used for amplification were: JWP1 (5’-GCACTGAAGTGACCAAGCGG-3’) and JWOPA7 (5’-GAAACGGGTG-3’), ERIC-2 (5’-AA GTAGTGACTGGGGTGAGCG-3’) and ERIC-1R (5’-ATGTAAGCTCCTGGGGATTCAC-3’) (Sosa and Zunino, 2013). For ERIC-PCR we used amplification conditions slightly different from Sosa and Zunino (2013), including an initial denaturation step at 94 °C for 5 min, followed by 45 cycles of 1 min at 94 °C, 1 min at 52 °C and 2 min at 72 °C, with a final extension at 72 °C for 10 min. The products of the reactions were analyzed by agarose gel electrophoresis, as previously explained.

To analyze the amplicons, a matrix with binary data (presence = 1 or absence = 0) was constructed for the quantification of genetic diversity using the GENES software (Cruz, 2008). Estimates of genetic similarity between each pair of lineages were obtained by means of the Dice similarity coefficient (Reif *et al.*, 2005), observing the interval of occurrence between one (putatively clones) to zero (highly divergent). A dendrogram was constructed using the matrix of dissimilarity estimated by unweighted paired group method of cluster analysis using arithmetic averages (UPGMA) (Cruz and Carneiro, 2003).

For ITS sequencing, after amplification of the 16S-23S intergenic regions (Angelos *et al.*, 2007), the amplicons were purified with kit MinElute® (Qiagen®). For Sanger sequencing, the reactions were carried out with kit BigDye Terminator v3.1 Cycle Sequencing, following the conditions laid down by the manufacturers. After the reaction, the generated fragments were subjected to capillary electrophoresis in a ABI 3500 Genetic Analyzer (Applied Biosystems). Consensus sequences were generated for each sample in DNA Baser (v.5.15) and aligned with sequences from GenBank (accesses: *Moraxella bovis* – CP030241.1; DQ647927.1; EU014535.1; EU014547.1 and EU014575.1, and *Moraxella bovoculi* – DQ153085.1; DQ153089.1 and DQ153093.1), and a phylogenetic tree was constructed by the Maximum Likelihood method using MEGA (v7.0.26).

In our study, the bacteria were isolated from around 10% of the herd, only from ill animals. Bacteria isolation prior to identification allows only viable bacteria to be detected. In addition, we focused only on hemolytic colonies, since the cytotoxin β-hemolysin is a known virulence factor to *M. bovis* (Postma *et al.*, 2008). We identified 13/28 *M. bovis,* and 15/28 *M. bovoculi,* from 90 animals, contradicting previous works that more frequently isolated *M. bovis* (O’Connor *et al.*, 2012). In our study, no *M. ovis* was isolated, while Schnee *et al.* (2015), who worked with *Moraxella* identification in pre-IBK, post-IBK, and acute IBK phase herds, observed *M. ovis* more frequently, but concluded that only *M. bovoculi* was directly related to the disease, since it was the only species with increased prevalence only in the acute IBK phase herd.

Using the PCR-derived techniques, the selected primers for RAPD (OPA-02, OPA-03, OPA-04, OPA-07, OPA-09, OPA-11 and OPA-13 – Table S1), JWP1-JWOPA07 and ERIC-PCR (PCR-derived techniques) generated together 107 analyzable amplicons ranging from six (OPA-07) to 19 (OPA-03) (Figure 1). Analyzable amplicons were used to generate a dendrogram (Figure 2) from a unique diversity matrix. Two main genetically differentiable groups (clades) were observed. As expected, the 14 *M. bovis* isolates remained in one clade, whereas the 17 *M. bovoculi* remained in the other (Figure 2). According to the calculated bootstrap values, the two clades have great stability, reaching values of 76.15% and 92.35% for *M. bovis* and *M. bovoculi*, respectively.

**Figure 1:**
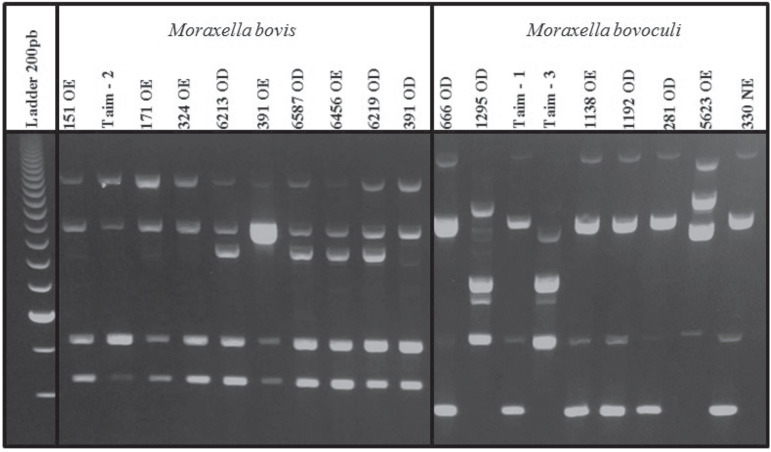
Example of RAPD technique using a single primer (OPA-03). After RAPD-PCR, ten samples of *M. bovis* (left side) and nine samples of *M. bovoculi* (right side) were run in an agarose gel and stained with ethidium bromide.

**Figure 2:**
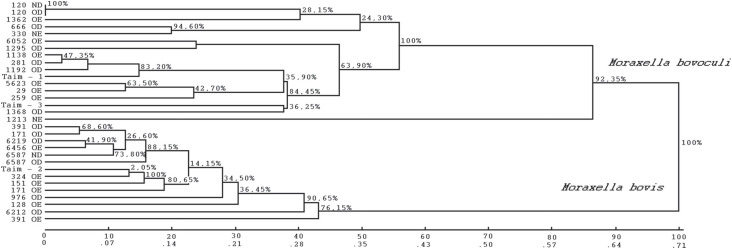
Dendrogram representing the genetic relationships between *M. bovis* and *M. bovoculi* isolates by the UPGMA method. Bootstrap values represented in the tree (2000 bootstrap repetitions).

Genetic distances between *M. bovis* samples ranged from 0.0379 to 0.4285, with a mean of 0.1970. The lowest values of genetic dissimilarity were recorded among the 391 OD and 171 OD isolates, collected from two distinct animals, but belonging to the same herd. On the other hand, samples 6213 OD and 391 OE were the genetically most distant, both collected from distinct animals of different municipalities.

The values of dissimilarities between *M. bovoculi* ranged from zero to 0.7297, with a mean of 0.3625. Among the samples studied, two are possibly clones (120 OD and 120 ND), what is not surprising, since they were sampled from the same animal (Figure 2). The highest index of genetic divergence was verified between the samples Taim-3 and 1213 NE, these being samples collected from different animals belonging to two farms located in different municipalities. Despite the low number of samples and the geographic proximity between collection areas, genetic diversity analyzed by PCR-derived techniques proved to be a satisfactory tool to reveal the existence of genetic variability/similarity among *Moraxella* spp. Except for one herd, we always isolated for both, *M. bovis* and *M. bovoculi,* strains with different genetic profiles from animals coexisting in the same area (data not shown), corroborating previous work that demonstrated genetic variability of *Moraxella* isolates within the herds (Conceição *et al.*, 2004).

By means of PCR-derived techniques, several previous studies have demonstrated genetic differences between isolates of *M. bovis* (Prieto *et al.*, 1999; Conceição *et al.*, 2004) or *M. bovis* and *M. bovoculi* (Sosa and Zunino, 2013) in Brazil, Argentina, and Uruguay, regions geographically related to the sampling of our study. The only previous study considering Brazilian isolates visualized a considerable higher number of possible clones than we did, possible due to the smaller number of analyzed amplicons (Conceição *et al.*, 2004).

We also constructed a phylogenetic tree based on the homology of the ITS sequence of the *Moraxella* spp. isolates (Figure 3). *M. bovis* and *M. bovoculi* were grouped into two different clades, corroborating with the dendrogram analysis (Figure 2). This technique was able to satisfactorily identify/differentiate the two species of the *Moraxella* genus; however it was less informative than PCR-derived techniques to reveal genetic differences between isolates. It produced a less branched tree, and the concordance between the different intraspecific clades (Figure 3) with those obtained by PCR techniques (Figure 2), was not comprehensive. This apparent inconsistency, nonetheless, could be due to differences in evolutionary pressure in different parts of genome. ITS sequencing was less effective in demonstrating genetic variation, probably because regions with significant interspecies variations but low intraspecific polymorphisms were sequenced, corroborating a previous study (Wang *et al.*, 2008). The higher discriminatory power of RAPD markers can be explained by the greater coverage of these markers throughout the whole genome. An alternative for increasing the discriminatory power of sequencing is to target additional regions of the bacterial genome within the same analysis.

**Figure 3:**
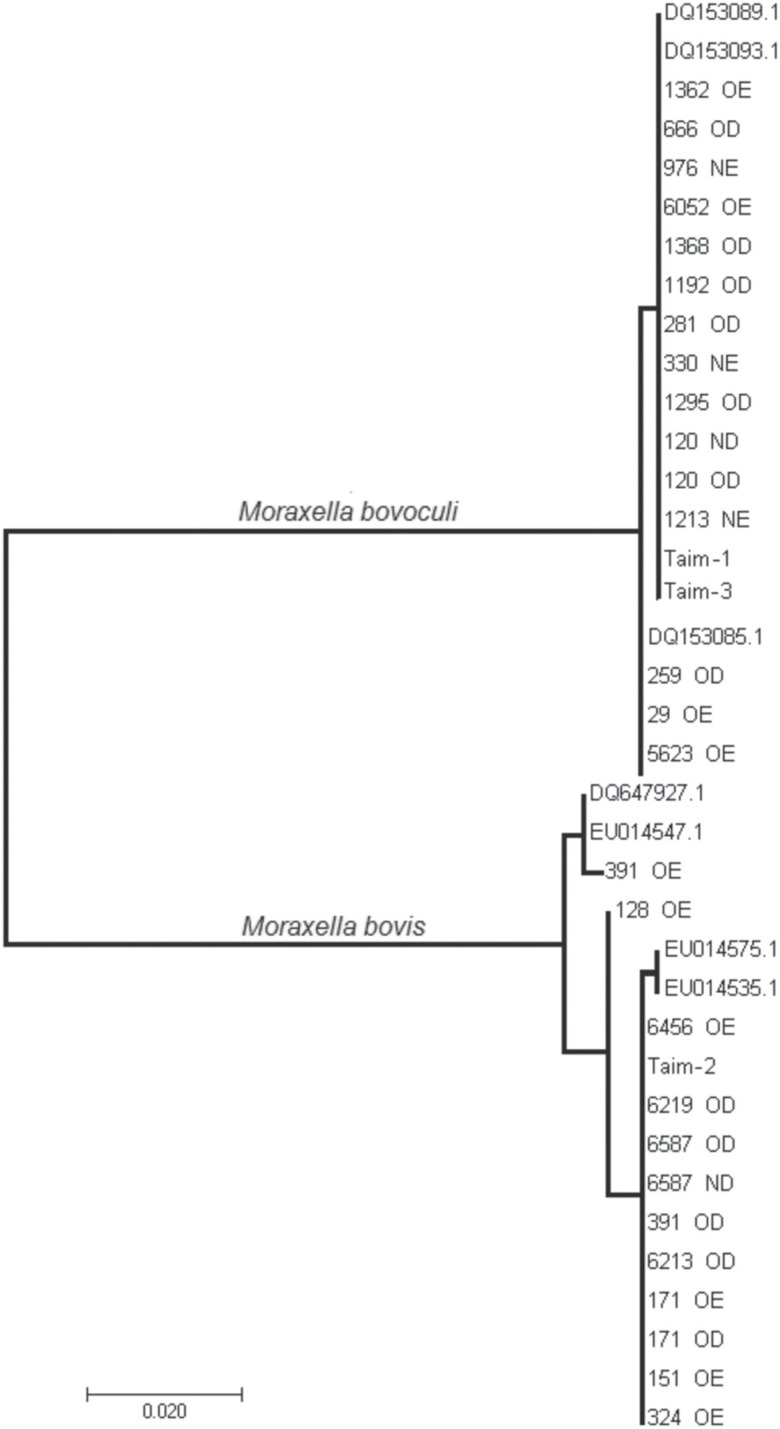
Phylogenetic tree of the ITS region of the *M. bovis* and *M. bovoculi* isolates inferred by the maximum likelihood algorithm.

Interestingly, three *M. bovis* pairs, each one isolated from the same animal (171 OD – 171 OE; 391 OD – 391 OE and 6587 OD – 6587 ND) were not genetically identical, when analyzed by PCR-derived techniques. These pairs showed genetics distance of 0.1304; 0.3333 and 0.1034, respectively (Figure 2). On the other hand, according to ITS sequencing, only the pair isolated from animal 391 diverged at 7 bp of the 656 bp analyzed (1.07%). This pair presented the highest distance when analyzed by PCR-derived techniques (0.3333). Considering only samples from animal 391, the 391 OD was more similar with the others collected in this work and with accesses EU014535.1 and EU014575.1 from GenBank, whereas the 391 OE sample was more similar with the GenBank samples EU014547.1 and DQ647927.1 (Figure 3). Consequently, samples 391 OD and 391 OE stayed in separate clades.

The co-infection of single animals with genetically distinct strains has not yet been documented for *M. bovis*, although for other bacteria this phenomenon was described (Ogle and Vasil, 1991; Gaspar *et al.*, 2011). This can be a complicating factor in the treatment of infections and in the efficacy of vaccines (Brown *et al.*, 1998; Cullen *et al.*, 2017), because different strains may have different antigenic and genetic properties, as well as distinct susceptibility profiles to antimicrobial (Gaspar *et al.*, 2011). We previously tested the antimicrobials susceptibility in these isolates and found no difference, however (Comin *et al.*, 2017).

Although there is circumstantial evidence of *M. bovoculi* involvement in the pathogenesis of IBK, it has not yet been possible to prove the participation in the etiology of the disease (Gould *et al.*, 2013). In our study, *M. bovoculi* was frequently isolated from animals with clinical cases, corroborating several previous studies (Angelos *et al.*, 2010; Libardoni *et al.*, 2012; Karthik *et al.*, 2018), and in some cases it was the only *Moraxella* species isolated from the herd. We also demonstrated a higher genetic difference for *M. bovoculi,* in comparison to *M. bovis,* which agrees with the recent findings that demonstrate, by sequencing, high genetic variability in this species (Dickey *et al.*, 2016).

Even with those evidences, currently the only commercial vaccine in Brazil includes only one strain of *M. bovoculi* among the antigens, while in the US, the vaccine contains eight serotypes. Studies showing correlation between genetic and antigenic variability are scarce. Conceição *et al.* (2004) showed low correspondence between these two parameters, for *M. bovis*. However, most recent studies involving the sequencing of cytotoxin from *M. bovis, M. bovoculi,* and *M ovis* showed genetic variability in the aminoacid sequence for this virulence factor, depending on the period of isolation (Farias *et al.*, 2015). Moreover, for *M. catharralis,* the head domains of UspA2/2H promotes a general evasion of the host immune system throughout the extensive sequence polymorphism in this protein (Su *et al.*, 2013). However, these two works did not correlate those differences with serological variability, nor with genetic diversity. Taken together, these findings suggest a possible variability in antigenic composition, in addition to genetic variation. Thus, we postulate that the high genetic variability can be translated as an antigenic variability, hampering the universality of vaccines.

Despite sampling geographically close regions, we demonstrated considerable genetic diversity in *M. bovis* and *M. bovoculi* strains, indicating that the species must present a corresponding antigenic diversity, which can negatively affect therapeutic choice, control measures, and mainly, vaccine efficiency. Moreover, we demonstrated for the first time that *M. bovis* genetically distinct strains can co-infecting the same animal, which can limit therapeutic and vaccine efficiency, even within a single farm.

## Supplementary material

The following online material is available for this article:

Table S1 RAPD primers.
