# Supplementary material for: Genetic differences among Moraxella bovis and Moraxella bovoculi isolates from infectious bovine keratoconjunctivitis (IBK) outbreaks in southern Brazil
Source: Genet Mol Biol. 2020 May 29;43(2):e20180380. doi: 10.1590/1678-4685-GMB-2018-0380 (PMC7271659; doi:10.1590/1678-4685-GMB-2018-0380)
Supplement: Supplementary file 1 [file 1415-4757-GMB-43-2-e20180380-s1.pdf]

Supplementary Material to "Genetic differences among *Moraxella bovis* and *Moraxella bovoculi* isolates from infectious bovine keratoconjunctivitis (IBK) outbreaks in southern Brazil"

Table S1 - RAPD primers. Number of analyzable bands for the RAPD Primers tested for diversity genetic *Moraxella* spp.

Primer RAPD	Samples			
	Taim - 1	Taim - 3	Taim -2	1213 NE
OPA 01	0	1	0	0
OPA 02	5	5	3	3
OPA 03	4	5	4	4
OPA 04	6	1	3	4
OPA 05	1	1	3	3
OPA 06	0	0	0	0
OPA 07	6	6	1	2
OPA 08	1	1	0	0
OPA 09	12	9	11	6
OPA 10	1	2	0	1
OPA 11	1	1	3	1
OPA 12	0	0	0	0
OPA 13	3	3	4	4
OPA 14	0	0	0	0
OPA 15	0	0	1	1
OPA 16	0	0	0	0
OPA 17	1	1	0	0
OPA 18	3	3	1	1
OPA 19	0	0	0	0
OPA 20	0	0	0	0
